# Supra Inguinal Fascia Iliac Versus PENG Block for Post-Operative Pain Management of Hip Arthroplasty: A Narrative Review

**DOI:** 10.3390/jcm14124050

**Published:** 2025-06-07

**Authors:** Shahab Ahmadzadeh, Megan S. Walker, Mary O’Dell Duplechin, Drake P. Duplechin, Charles J. Fox, Sahar Shekoohi, Alan D. Kaye

**Affiliations:** 1 Department of Anesthesiology, Louisiana State University Health Sciences Center Shreveport, Shreveport, LA 71103, USA; 2 School of Medicine, Louisiana State University Health Sciences Center at New Orleans, New Orleans, LA 70112, USA; 3 School of Medicine, Louisiana State University Health Sciences Center Shreveport, Shreveport, LA 71103, USA; mmo002@lsuhs.edu (M.O.D.); dpd001@lsuhs.edu (D.P.D.); 4 Departments of Anesthesiology and Pharmacology, Toxicology, and Neurosciences, Louisiana State University Health Sciences Center Shreveport, Shreveport, LA 71103, USA

**Keywords:** analgesics, fascia iliac block, nerve block, arthroplasty, hip, pain, post-operative

## Abstract

Effective post-operative pain management following hip arthroplasty is critical to improving recovery, reducing opioid consumption, enhancing mobility, and reducing the risk of complications for patients. Multimodal anesthesia strategies, including the supra inguinal fascia iliac block (SIFIB) and the periarticular nerve group (PENG) block have become the new point of focus as opposed to traditional methods previously used. This narrative review compares the SIFIB and the PENG block in their efficacy to treat post-operative pain management. Mechanism of action, safety, patient outcomes, and clinical applications are compared between the two blocks for evaluation. Clinical studies have indicated that both blocks reduce post-operative pain and reduce opioid use. In contrast, SIFIB has shown to be more preferred in more complex procedures such as total hip arthroplasty, which requires extensive nerve coverage despite its longer onset time. The SIFIB has been shown to carry a higher risk of impairing motor function, making the PENG highly preferred in patients where quick mobility improvement is prioritized. The PENG block also showed slightly higher efficacy in reducing pain associated with post-operative passive limb movements, and a slight decrease in opioid consumption in comparison to SIFIB in the early post-operative time frame. Although the PENG shows more benefits in the early stages of post-operative recovery, the SIFIB shows similar outcomes to PENG over longer durations of recovery. Future studies can aid in establishing a framework for tailoring block selection to individual patient needs to optimize clinical outcomes.

## 1. Introduction

Hip arthroplasty surgeries can be associated with immense amounts of post-operative pain, which can have a negative outcome on a patient’s quality of life, longer hospital stay, delayed recovery, and increased opioid consumption, making post-operative pain management a critical aspect following these surgeries [1]. With recent medical developments, traditional post-operative treatment has seen efforts to move away from opioid medications and towards long-acting analgesics that effectively reduce pain, increase mobility, limit opioid use, and minimize complications [2]. Two recent developments in multimodal anesthesia are the supra inguinal fascia iliac block (SIFIB) and the periarticular nerve group (PENG) block. Both techniques have shown promise in improving post-operative outcomes for hip arthroplasty patients, and their roles continue to evolve within the framework of multimodal analgesia [3].

Both the SIFIB and PENG blocks are increasingly being used as part of multimodal anesthesia strategies. Multimodal anesthesia uses a combination of drugs to achieve a variety of different mechanisms of action to obtain the synergistic effect of the goal. These agents aim to reduce pain through blocking multiple pathways while limiting opioid consumption. Incorporating regional techniques like these allows physicians to address both somatic and articular pain transmitted by the nerves targeted in these blocks, while aiding in a quicker recovery and higher quality of patient outcomes [1].

The supra inguinal fascia iliac block provides a greater area of relief due to the injection site being located above the inguinal ligament. This allows the analgesic to enhance the blockage of the femoral nerve as well as other branches that run through the iliaca compartment but sparing the obturator nerve. SIFIB has proven to be efficacious in improving pain outcomes post-operatively [4,5]. While effective for post-operative pain relief, one drawback is the risk of quadriceps weakness related to partial motor blockade, which may hinder early mobilization [6].

The periarticular nerve block targets joint-related branches of the femoral nerve and the accessory obturator nerve and has been seen to improve pain post-operatively while limiting the consequences on motor function prior to hip arthroplasty procedures. This approach has a more localized target of the anterior hip capsule in comparison to the SIFIB, which has a larger area of action. The PENG block has been recognized for its facilitation in post-operative mobilization and rehabilitation in earlier stages following the hip arthroplasties compared to other agents such as non-steroidal inflammatory drugs, systemic opioids, and lumbar plexus blocks, which have traditionally been used in these procedures [3,7].

The aim of the present investigation, therefore, is to compare the supra inguinal fascia iliac block and periarticular nerve block and their components in being used for post-operative management of a hip arthroplasty, with a focus on their mechanisms of action, clinical outcomes, safety profiles, and role in multimodal analgesia. While both techniques have demonstrated efficacy, this review seeks to explore their differences and assess whether one should be preferred over the other in clinical practice.

## 2. Mechanisms of Action of Supra Inguinal Fascia Iliac Block and PENG Block

Post-operative pain management is critical in hip arthroplasty to improve patient comfort, facilitate early mobilization, and reduce post-operative opioid consumption. Two regional anesthesia techniques gaining traction are the supra-inguinal fascia iliaca block and the pericapsular nerve group block. While both target the innervation of the hip joint, they do so through distinct anatomical approaches and mechanisms.

### 2.1. Anatomical Considerations

**Figure 1:**
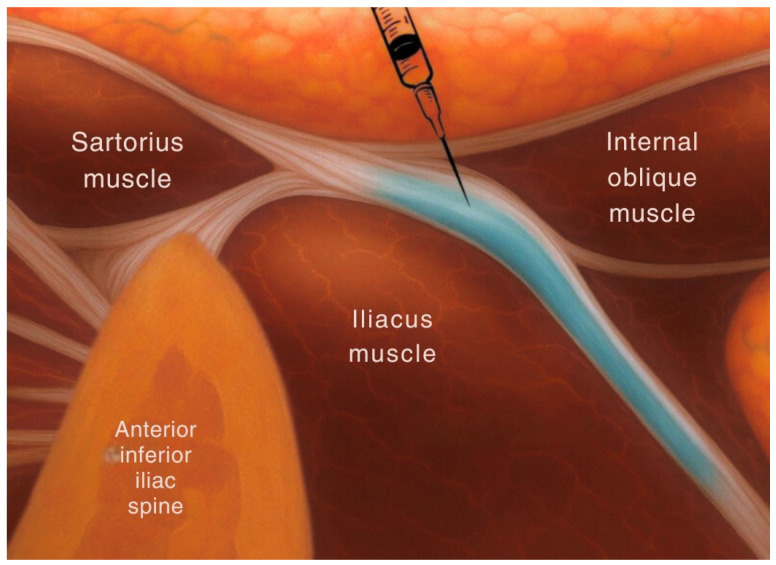
Anatomical location of the SIFI block.

**Figure 2:**
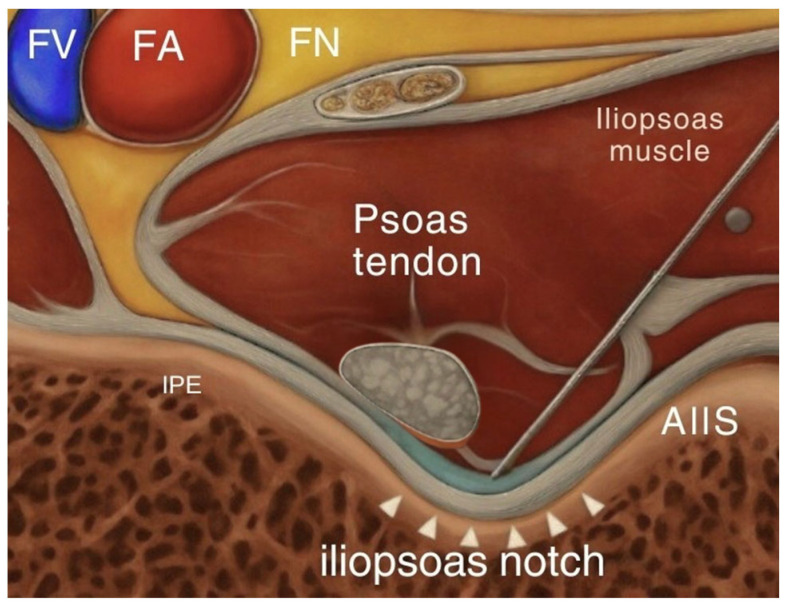
Anatomical location of PENG block.

The supra-inguinal fascia iliaca block targets the anterior branches of the lumbar plexus, providing anesthesia to the femoral nerve, lateral femoral cutaneous nerve, and obturator nerve. These nerves supply sensation to the anterior hip capsule, femoral shaft, and areas commonly involved in surgical incisions [8]. This block delivers local anesthetic above the inguinal ligament, deep to the fascia iliaca (Figure 1). By reaching these branches proximally, the SIFI block provides a broader coverage compared to infra-inguinal blocks, making it more effective for pain following hip surgery. In contrast, the pericapsular nerve group block involves injecting local anesthetic into the fascial plane located between the psoas tendon and the superior pubic ramus medial to the anterior inferior iliac spine (Figure 2). The goal of this interfascial plane block is to target the articular branches of the femoral, obturator, and accessory obturator nerves [7]. By focusing on the sensory branches that directly innervate the hip joint, the PENG block aims to provide targeted analgesia without significantly affecting motor function, which facilitates early post-operative ambulation.

### 2.2. Pharmacologic Profiles

From a pharmacological standpoint, both blocks commonly use long-acting local anesthetics such as bupivacaine or ropivacaine. These agents share similar mechanisms of action, primarily by blocking voltage-gated sodium channels, preventing depolarization and nerve transmission. However, they differ in some of their pharmacodynamic and pharmacokinetic profiles. Bupivacaine has a longer duration of action and higher potency. Bupivacaine works by binding to sodium channels within neurons intracellularly, thereby inhibiting the influx of sodium ions and preventing the depolarization process. As an amide group local anesthetic, it is metabolized mainly in the liver through glucuronic acid conjugation.

Despite its clinical effectiveness, the use of racemic bupivacaine has been associated with adverse effects in some individuals, including toxicity affecting the heart and central nervous system [9]. Ropivacaine, while slightly less potent, has a better safety profile and causes less motor blockades at equivalent doses. Ropivacaine has lower lipophilicity compared to bupivacaine, making it less likely to infiltrate large, myelinated motor fibers, which leads to a milder motor block. It offers improved sensory–motor separation, with a preferential effect on pain-conducting Aδ and C fibers, while sparing the Aβ fibers that play a key role in motor activity [9]. This makes it preferable for procedures where motor function preservation is desired, such as with PENG blocks. The onset time and duration can vary slightly between the two agents and are also influenced by the vascularity of the injection site. The supra-inguinal fascia iliaca block, given its more proximal location and larger spread, may have a slightly delayed onset but longer duration due to the slower systemic absorption. PENG blocks, though more targeted, are performed in a highly vascular area and may have a quicker onset but potentially shorter duration if not supplemented with adjuncts.

## 3. Benefits and Risks of Supra Inguinal Fascia Iliaca Block and PENG Block

While these blocks aim to minimize opioid requirements, enhance post-operative recovery, and facilitate early mobilization, each technique comes with certain contraindications and considerations. Balancing the benefits of pain relief with the risks of complications is essential when choosing the appropriate technique for individual patients.

The supra inguinal fascia iliaca block offers extensive coverage that results in effective analgesia, particularly useful for major hip and proximal femur procedures. Clinical studies have shown that patients receiving the supra inguinal fascia iliaca block reach physical therapy ambulation goals faster than those receiving other anesthetic methods, such as a lumbar epidural, while also using fewer opioids post-operatively [10]. Additionally, patients undergoing the supra inguinal fascia iliaca block have reported lower post-operative pain scores at rest, indicating better pain control during recovery [11]. As stated previously, one of the benefits of the supra inguinal fascia iliaca block is its longer duration of analgesia due to proximal spread and slower systemic absorption. This extended analgesia is particularly valuable for patients who require sustained pain management throughout their post-operative recovery, especially those undergoing more complex surgeries. However, this broader spread also increases the risk of inadvertent motor blockade, which can hinder early ambulation. While complications have rarely been observed, there is a possibility that vascular puncture, local anesthetic systemic toxicity, and femoral nerve motor blockade can occur. Due to the injection being in a relatively deep and vascular-rich area, ultrasound guidance is essential to minimize risks.

The pericapsular nerve group block is a newer technique and offers a more selective approach to analgesia by providing effective pain relief to the anterior hip capsule without significantly affecting motor function. Studies have shown that patients receiving pericapsular nerve group blocks have decreased post-operative opioid use compared to those receiving fascia iliaca blocks [12]. This makes the pericapsular nerve group block an appealing option for patients who require motor function preservation for early mobilization. Additionally, PENG blocks have been associated with better pain control scores, particularly for patients undergoing hip surgeries [13]. While the PENG block has a more favorable motor-sparing profile, it still has risks that should be considered. The injection site lies near the femoral artery and iliopsoas tendon, and improper technique could result in vascular puncture, hematoma, or local anesthetic spreading to unintended neural structures. Since the PENG block is relatively new, research on its long-term safety and complication rates is still ongoing. Both blocks are contraindicated in patients with allergies to local anesthetics, infection at the injection site, significant anatomical variations, coagulopathy or on anticoagulants, pregnancy, poor patient cooperation, or scarring from previous surgeries in the area.

Thus, both the supra inguinal fascia iliaca and PENG blocks have shown significant benefits in enhancing post-operative recovery by providing superior pain relief and reducing opioid consumption. However, each block comes with its own safety considerations and adverse effects like motor weakness or vascular puncture. Careful patient selection is essential for optimizing the benefits while minimizing the risks. Ongoing research will further clarify the safety profiles, complications, and long-term benefits of these techniques in different patient populations.

## 4. Clinical Applications of Supra Inguinal Fascia Iliac Block and PENG Block

The supra-inguinal fascia iliaca block and the pericapsular nerve group block have both seen increasing use in hip arthroplasty surgeries, offering advantages over older approaches. Understanding how these blocks are used in different types of hip surgeries and comparing them to older methods highlights their growing importance. They play a key role in enhancing recovery times and improving overall patient outcomes.

### 4.1. Total Hip Arthroplasty

In total hip arthroplasty, the supra-inguinal fascia iliaca block has largely replaced traditional lumbar plexus and femoral nerve blocks. Historically, lumbar plexus blocks were favored for their extensive nerve coverage, but they often caused significant motor block, impairing early mobility and recovery. The femoral nerve block also provided good analgesia but left a portion of the hip joint inadequately covered, especially the posterior hip capsule. The supra-inguinal fascia iliaca block is more refined, targeting both the femoral and lumbar nerves through a single injection, which provides comprehensive analgesia while preserving motor function, particularly hip flexion and extension. This targeted approach reduces opioid use, enhances recovery times, and minimizes complications related to motor impairment, such as difficulty in ambulation post-surgery.

### 4.2. Hemiarthroplasty

In hemiarthroplasty, the PENG block is becoming increasingly popular, particularly for its ability to offer motor-sparing analgesia. Traditional blocks like lumbar plexus and femoral nerve blocks were commonly used, but they offered less precise analgesia, and patients often experienced motor weakness. The PENG block, in contrast, focuses on the anterior hip capsule and hip joint, which is where the pain is most prominent in hemiarthroplasty. By focusing on the articular branches of the hip, the PENG block significantly improves pain management without hindering muscle function, making it an ideal choice for these patients.

### 4.3. Hip Resurfacing Arthroplasty

Unlike total hip replacement, where both the acetabulum and the femoral head are replaced, hip resurfacing arthroplasty involves removing the damaged femoral head and capping the femoral neck with a metal prosthesis, leaving most of the proximal femur intact [14]. The acetabulum is still resurfaced with a metal cup. Hip resurfacing benefits from the use of the PENG block. Historically, femoral nerve and sciatic blocks were used to provide analgesia in hip resurfacing procedures, but they often resulted in motor block and discomfort, particularly in the hip’s anterior and posterior regions. The PENG block provides a more targeted approach, covering the anterior hip capsule where most of the pain originates during hip resurfacing. This allows for effective pain relief while minimizing motor blockade, which is crucial for patients undergoing hip resurfacing, as they often benefit from a quicker return to mobility.

### 4.4. Revision Hip Arthroplasty

In revision hip arthroplasty, the supra-inguinal fascia iliaca block is a great option due to its broad nerve coverage and comprehensive pain relief. Revision surgeries tend to be more complex, with more extensive tissue damage, and therefore require a more robust anesthetic approach. Traditional blocks like lumbar plexus and femoral nerve blocks offer partial coverage, potentially leaving patients with incomplete pain relief, particularly in the posterior hip. The supra-inguinal fascia iliaca block provides extensive analgesia by targeting the lumbar plexus, femoral nerve, and other related structures, ensuring that pain relief is maximized throughout the hip region. Additionally, the supra-inguinal fascia iliaca block is associated with less motor impairment when compared to lumbar or femoral blocks.

The clinical use of supra-inguinal fascia iliaca block and PENG blocks represents a significant advancement over older nerve blocks, such as lumbar plexus and femoral nerve blocks, which often left patients with limited motor function and increased opioid requirements. Both the supra-inguinal fascia iliaca block and PENG blocks offer enhanced control over pain while minimizing the risk of motor weakness, which is crucial for promoting faster recovery and improving mobility post-surgery. Both blocks have demonstrated a reduction in opioid consumption, contributing to improved patient outcomes and a lower risk of opioid-related complications. In conclusion, both the supra-inguinal fascia iliaca and PENG blocks offer substantial improvements in pain management following hip arthroplasty (Table 1).

## 5. Comparison of Patient Outcomes Using Supra Inguinal Fascia Iliac Block and PENG Block

Effective post-operative analgesia is crucial in hip arthroplasty, particularly related to the need for early mobilization, minimizing opioid-related side effects, and optimizing recovery. Regional anesthesia techniques have emerged as favorable alternatives to systemic analgesia and central neuraxial blocks. Among these, the supra-inguinal fascia iliac compartment (SFIC) block and the pericapsular nerve group (PENG) block have become increasingly relevant, each targeting different elements of hip joint innervation.

The SFIC block, introduced by Hebbard et al., enables anesthetic spread above the inguinal ligament and within the fascia iliac compartment, affecting the femoral nerve (FN), lateral femoral cutaneous nerve (LFCN), and obturator nerve (ON) [15]. However, it may spare the accessory obturator nerve (AON) and can impair quadriceps strength, posing challenges for early mobilization. In contrast, the PENG block, introduced in 2018, directly targets the articular branches of FN, ON, and AON, sparing motor function by avoiding main nerve trunks [16].

Multiple studies have compared these blocks across key post-operative parameters. Gonabal et al. (2024) randomized 66 patients to receive either SFIC or PENG block following hip or proximal femur surgery [17]. At 24 h post-op, PENG patients had significantly lower VAS pain scores during movement (*p* = 0.018) and stronger quadriceps power (*p* = 0.001). Opioid use was also lower in the PENG group (28.5% vs. 71.4% receiving morphine equivalents, *p* = 0.03). Post-operative cognitive dysfunction rates were similar.

Jadon et al. (2021) also compared SFIC vs. PENG block for ease of positioning during spinal anesthesia [18]. They found that PENG block significantly improved ease of spinal positioning (EOSP score 2.15 vs. 1.39, *p* < 0.0001), suggesting superior analgesia in the preoperative setting. While both blocks lowered pain scores from baseline, PENG provided more consistent relief at rest and during passive limb movement. Tramadol use and time to first analgesia were not significantly different, but PENG showed a better median pain profile over 24 h.

In another clinical trial, Choi et al. (2022) investigated outcomes after total hip arthroplasty (THA) under general anesthesia [2]. Pain scores at rest were lower at 6 and 24 h in the PENG group, although no difference was detected by 48 h. Notably, quadriceps strength preservation was equivalent between groups, supporting PENG’s selective sensory blockade. Opioid consumption over 48 h showed no significant difference, but patients receiving PENG had a slight trend toward reduced use.

Finally, another study comparing SFIC and PENG block specifically for THA patients reinforced these findings. Patients with PENG block reported better early post-operative pain control and improved comfort during physiotherapy initiation, though long-term differences diminished over time [19].

Taken together, these findings suggest that PENG block may offer improved immediate post-operative outcomes in terms of pain control, patient comfort, and quadriceps preservation—an essential factor for rehabilitation. Complication rates were observed to be higher in the PENG group with 15% while 8.3% was recorded in the SFIC block group [20]. While both blocks are viable, PENG may support faster initiation of physical therapy and higher patient satisfaction in the critical 24 h post-operative window. This comparison underscores the growing favorability of PENG block in hip arthroplasty.

## 6. Discussion

The growing interest in regional anesthesia techniques for hip arthroplasty reflects a broader shift toward optimizing perioperative pain control while minimizing opioid use and enhancing recovery. Both the supra-inguinal fascia iliac compartment (SFIC) block and the pericapsular nerve group (PENG) block have demonstrated benefits, but they come with unique advantages and limitations that may influence their adoption in clinical practice.

One of the primary advantages of the PENG block is its motor-sparing profile. By targeting only the articular branches of the femoral, obturator, and accessory obturator nerves, it preserves quadriceps strength, which is critical for early ambulation and participation in physical therapy. In contrast, the SFIC block—though effective in providing analgesia—often affects motor function due to its broader nerve distribution, including the femoral nerve trunk. This difference can have significant downstream effects on mobility and fall prevention in post-operative patients.

Cost-effectiveness is another important consideration. While both blocks can be performed under ultrasound guidance using similar equipment and anesthetic agents, the PENG block’s selective approach may lead to fewer complications and shorter rehabilitation time, which may reduce overall healthcare costs. However, real-world data on this economic impact remain limited. Availability of trained personnel and institutional familiarity may also skew preference toward the more established SFIC block in some settings.

From an efficacy standpoint, studies have shown mixed results over longer time frames. While PENG block may offer superior analgesia in the first 24 h, differences tend to diminish by 48 h. Additionally, opioid consumption between the two blocks evens out over time. This suggests that the immediate post-operative period is where PENG block provides the most value especially in enhanced recovery after surgery protocol (ERAS). Studies suggest PENG may benefit up to 90% of patients compared to the SFIC block which benefited up to 75% of patients [21,22,23,24]. Nevertheless, SFIC block remains a dependable and broader-coverage option, especially when patient variability in nerve anatomy is a concern.

Safety-wise, both blocks have favorable profiles, with low incidence of complications reported across multiple trials. However, due to the deeper location and proximity to vascular structures in the PENG block, a slightly higher level of expertise may be required to ensure safe administration. As such, widespread adoption may be limited by training opportunities and comfort level among anesthesiologists.

In the future, there are several key areas for future research. Long-term outcomes comparing functional recovery, quality of life, and rehabilitation milestones between these two blocks are needed. Additionally, their use in other orthopedic surgeries—such as femur fracture repair or acetabular procedures—could broaden the utility of these techniques. Comparative effectiveness trials involving other regional blocks like the lumbar plexus block or quadratus lumborum block may also help define best practices.

As technology advances, integration of nerve stimulation or artificial intelligence enhanced ultrasound may improve precision and outcomes for both PENG and SFIC blocks. Understanding how these modalities perform in high-risk populations, such as the elderly or those with cognitive impairment, may further inform decision-making and guidelines for block selection.

**Figure 3:**
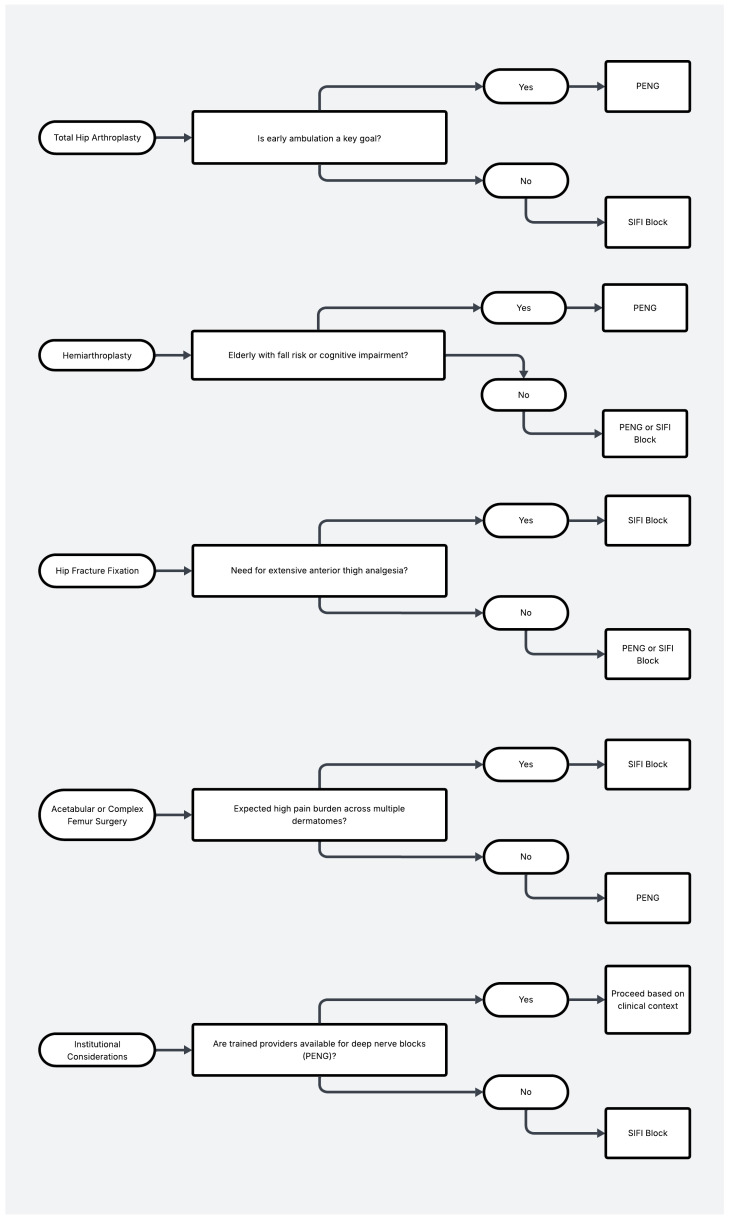
Preferred block type based on patient priorities.

In summary, the PENG block appears to be a highly promising alternative to the SFIC block in hip arthroplasty, particularly when early mobility and motor preservation are prioritized. However, both blocks remain viable and effective tools, and their selection should ultimately be tailored to institutional capabilities, patient characteristics, and surgical goals (Figure 3).

This study is subject to several limitations. First, potential heterogeneity in block performance—including operator experience, anatomical variability, and differences in local anesthetic concentration or volume—may have influenced outcomes. Future studies should aim for standardized, reproducible techniques with clear procedural protocols. Second, the adjunct analgesic regimen was not fully controlled, and variability in intraoperative and post-operative medications may have confounded the observed differences in VAS scores and opioid consumption. A more uniform multimodal analgesia approach across cohorts would enhance interpretability. Lastly, the study’s limited follow-up period restricts insight into long-term functional recovery, warranting further research with extended observation [25,26].

## 7. Conclusions

The present investigation compares the supra-inguinal fascia iliaca block (SIFIB) and the pericapsular nerve group (PENG) block, both of which have demonstrated efficacy in managing post-operative pain. As the shift moves away from traditional techniques toward multimodal anesthesia, their efficacy is examined by exploring mechanisms of action, clinical outcomes, safety profiles, enhanced recovery, and reduced opioid consumption. We identified the advantages and limitations of each technique.

SIFIB provides broad analgesic coverage by targeting the femoral, lateral cutaneous, and obturator nerves, making it effective in complex procedures requiring extensive nerve blockade. Its long duration of action ensures adequate pain control and reduced opioid use compared to traditional methods. Conversely, it may impair motor function, posing challenges for early mobilization.

The PENG block is a newer technique targeting the articular branches of the femoral, obturator, and accessory obturator nerves while avoiding major motor branches. It offers significant pain control while preserving motor function and is highly effective in hip arthroplasty where early mobility is crucial.

Studies comparing SIFIB and PENG show that PENG correlates with reduced opioid consumption and pain scores post-operatively, while maintaining quadriceps strength. One study found PENG more effective in reducing pain at rest and during limb movement. Although outcomes between SIFIB and PENG converge over time, PENG appears more effective in early post-operative pain control, patient satisfaction, and mobility. However, it requires greater proficiency due to anatomical complexity. SIFIB is favored for its long duration and broader coverage, though it carries a risk of motor impairment.

In conclusion, both SIFIB and PENG represent significant advances in post-operative pain management for hip arthroplasty. Despite promising findings, further research is needed to support a patient-centered approach in block selection to optimize outcomes.

## Figures and Tables

**Table 1:** Clinical applications of the supra-inguinal fascia iliaca block and pericapsular nerve group block.

Type of Hip Arthroplasty	Previous Blocks Used	Preferred Block	Comparison of Blocks
Total Hip Arthroplasty	Lumbar plexus block and femoral nerve block	The Supra Inguinal Fascia Iliaca (SIFI)	SIFI offers more targeted pain relief with less motor block, improving post-operative mobility and recovery while still providing effective coverage compared to the femoral and lumbar plexus blocks. SIFI provides complete joint coverage while PENG covers primarily the anterior hip.
Hemiarthroplasty	Lumbar plexus block and femoral nerve block	The Pericapsular Nerve Group (PENG) block	PENG is ideal for providing targeted anterior hip capsule analgesia with less motor block, offering effective pain relief while preserving muscle function for early mobilization.
Hip Resurfacing Arthroplasty	Femoral nerve block and sciatic block	The Pericapsular Nerve Group (PENG) block	PENG would be ideal since it targets the anterior hip capsule effectively, where most of the pain from hip resurfacing would originate.
Revision Hip Arthroplasty	Lumbar plexus block and femoral nerve block	The Supra Inguinal Fascia Iliaca (SIFI)	SIFI provides comprehensive pain relief across both anterior and posterior hip regions, with less motor block compared to lumbar and femoral blocks, facilitating improved recovery and mobility in revision surgeries.
